# Chemogenetic and pharmacological activation of the oxytocin system ameliorates adjuvant arthritis in rats

**DOI:** 10.3389/fendo.2026.1941424

**Published:** 2026-10-30

**Authors:** Wataru Hashimoto, Hitoshi Suzuki, Hiroaki Fujihara, Hiromichi Ueno, Hajime Fukushima, Rie Ariyoshi, Manabu Tsukamoto, Makoto Kawasaki, Tomoya Arashi, Takafumi Horishita, Akinori Sakai, Yoichi Ueta, Takashi Maruyama

**Affiliations:** 1 Department of Anesthesiology, School of Medicine, University of Occupational and Environmental Health, Kitakyushu, Japan; 2 Department of Orthopaedic Surgery, School of Medicine, University of Occupational and Environmental Health, Kitakyushu, Japan; 3 Department of Physiology, School of Medicine, University of Occupational and Environmental Health, Kitakyushu, Japan

**Keywords:** adjuvant arthritis, chemogenetics, DREADD, inflammation, LIT-001, nociceptive pain, oxytocin, oxytocin receptors

## Abstract

Oxytocin (OXT) is an endogenous neuropeptide involved in the regulation of nociceptive processing and inflammatory responses. Chronic inflammatory arthritis is characterized by persistent inflammation and pain, and understanding neuroendocrine mechanisms that regulate both processes may provide new insights into the pathophysiology of chronic inflammatory pain. Previous studies have shown that endogenous OXT has both anti-inflammatory and antinociceptive effects, suggesting that activation of the OXT system may influence arthritis by modulating inflammatory and pain-related pathways. However, whether activation of endogenous OXT signaling can modulate the progression of chronic inflammatory arthritis and its associated pain remains incompletely understood. Therefore, this study investigated whether chemogenetic activation of OXT neurons and pharmacological stimulation of OXT receptors modulate disease progression and pain-related outcomes in a rat model of adjuvant arthritis. Adjuvant arthritis was induced in adult male OXT-hM3Dq-mCherry transgenic rats (n = 39) and adult male Wistar rats (n = 10). The transgenic rats were treated with CNO, whereas Wistar rats were treated with LIT-001, with the corresponding vehicle-treated groups serving as controls. Arthritis severity, ankle swelling, mechanical hypersensitivity, joint histopathology, and neuronal activity markers were evaluated. Chemogenetic activation of OXT neurons attenuated arthritis progression during both the onset phase (Days 10-12) and established phase (Days 15-17). During the onset phase, it also reduced ankle swelling and prevented the decline in mechanical nociceptive thresholds. Descriptive histological evaluation showed less pronounced inflammatory cell infiltration, synovial hyperplasia, pannus formation, cartilage destruction, and bone erosion in affected joints. Similarly, repeated administration of the oxytocin receptor agonist LIT-001 ameliorated arthritis severity, joint swelling, and inflammatory pain-related behaviors. Immunohistochemical analyses further revealed increased FosB expression in the paraventricular nucleus and reduced numbers of FosB-immunoreactive and tryptophan hydroxylase (TPH)-immunoreactive cells in the dorsal raphe nucleus following LIT-001 treatment. These findings show that chemogenetic activation of endogenous OXT neurons and administration of the OXT receptor agonist LIT-001 ameliorate arthritis pathology and inflammatory pain-related behaviors in adjuvant arthritis. The OXT system may therefore contribute to the regulation of chronic inflammatory arthritis through coordinated effects on peripheral inflammation and central pain processing.

## 1 Introduction

Oxytocin (OXT) is a neuropeptide synthesized primarily in the paraventricular nucleus (PVN) and supraoptic nucleus (SON) of the hypothalamus. OXT is released from the posterior pituitary into the systemic circulation and plays essential roles in parturition and lactation. In addition to its peripheral endocrine functions, OXT also acts within the central nervous system and contributes to pain modulation (1). Thus, OXT may function not only as a peripheral hormone but also as a neuroendocrine regulator involved in chronic pain and inflammatory diseases.

The OXT system modulates pain through multiple pathways. OXT neurons originating in the PVN project to the spinal dorsal horn and brainstem, where they influence nociceptive processing (2). Activation of the PVN or the OXT system produces antinociceptive effects in animal models (3, 4). Moreover, administration of OXT or OXT receptor agonists attenuates pain-related behaviors in models of neuropathic, inflammatory, and visceral pain. Together, these findings indicate that the OXT system constitutes an endogenous mechanism for pain regulation (5–7).

Accumulating evidence also suggests a role for the OXT system in the regulation of inflammatory responses. In acute inflammatory models, endogenous OXT suppresses both nociceptive and inflammatory responses (8). Altered OXT receptor expression has been reported in inflamed tissues and immune cells, suggesting that OXT may modulate inflammatory pathology through neuroimmune interactions (9–12). However, it remains unclear whether activation of the endogenous OXT system can attenuate not only pain-related behaviors but also arthritis severity and joint tissue damage in chronic inflammatory arthritis.

Adjuvant arthritis (AA) in rats is a well-established model of chronic inflammatory pain characterized by arthritis, mechanical hypersensitivity, and osteochondral damage (13, 14). Previous studies have demonstrated activation of both the hypothalamic–posterior pituitary and hypothalamic–spinal OXT pathways in AA rats (11, 15). These findings suggest that the OXT system serves as an endogenous protective mechanism against AA pathology. However, whether further enhancement of this response can suppress arthritis progression, pain-related behaviors, and joint tissue damage remains unknown.

In the present study, we hypothesized that activation of the endogenous OXT system may modulate chronic inflammatory arthritis through effects on both peripheral inflammatory responses and pain processing. We therefore investigated whether activation of the endogenous OXT system modulates arthritic symptoms, mechanical hypersensitivity, and joint tissue damage in AA rats. We previously demonstrated, using OXT-hM3Dq-mCherry transgenic rats, that activation of endogenous OXT neurons contributes to pain modulation (8, 16). Here, we applied a chemogenetic approach using Designer Receptors Exclusively Activated by Designer Drugs (DREADDs) to the AA model and evaluated the effects of OXT neuron activation on arthritis progression, pain-related behaviors, and joint tissue damage. In addition, we analyzed neuronal activity markers in the PVN, SON, and dorsal raphe nucleus (DR) following administration of the non-peptide OXT receptor agonist LIT-001 to investigate central neural responses associated with OXT receptor activation.

## 2 Materials and methods

### 2.1 Animals

Adult male OXT-hM3Dq-mCherry transgenic (Tg) rats (heterozygous; n = 39, 8 weeks old, 240–310 g) and adult male Wistar rats (n = 10, 6 weeks old, 170–200 g) were used. The Tg rat line was maintained by breeding with Wistar rats. Animals were housed under standard laboratory conditions (24 ± 1 °C, 55 ± 5% humidity, 12-h light/dark cycle, lights on at 07:00) with free access to standard laboratory chow and water.

### 2.2 Ethics statement

All experimental procedures were approved by the Animal Care and Use Committee of the University of Occupational and Environmental Health, Japan (AE10-012), and were conducted in accordance with the Guiding Principles for the Care and Use of Animals approved by the Physiological Society of Japan.

### 2.3 Induction of adjuvant arthritis

Adjuvant arthritis (AA) was induced on Day 0 by intradermal injection of 1 mg heat-killed *Mycobacterium tuberculosis* (InvivoGen, San Diego, CA, USA) suspended in liquid paraffin (Nacalai Tesque, Kyoto, Japan) into the base of the tail of OXT-hM3Dq-mCherry Tg rats and Wistar rats (17).

After AA induction, arthritis severity was assessed daily using an arthritis index. The severity of arthritis was scored for each limb based on the degree of erythema, swelling, and joint deformity as follows: 0, no erythema or swelling; 1, slight erythema or swelling in one digit; 2, erythema and swelling in two or more digits; 3, erythema and swelling of the ankle joint; and 4, severe erythema and swelling involving the digits and ankle joint with loss of ankle flexion (18). Scores from all four limbs were summed to obtain the arthritis index for each animal (maximum score, 16). Assessments were performed independently by two observers.

### 2.4 Experimental protocols

Three independent experiments were performed. In Experiments 1 and 2, OXT-hM3Dq-mCherry Tg rats were allocated to CNO-treated or vehicle-treated groups based on body weight to minimize differences in body weight between the groups; vehicle-treated Tg rats served as the control groups and did not receive CNO. Experiment 1 examined the effects of CNO administration during the established phase of arthritis (Days 15–17), whereas Experiment 2 examined its effects during the onset phase (Days 10–12). Experiment 3 used Wistar rats assigned to LIT-001-treated or vehicle-treated groups. The experimental schedule and group sizes are summarized in **Figure 1**. The evaluators were not blinded to treatment allocation during the assessment of arthritis scores, mechanical nociceptive thresholds, or histopathological findings.

**Figure 1:**
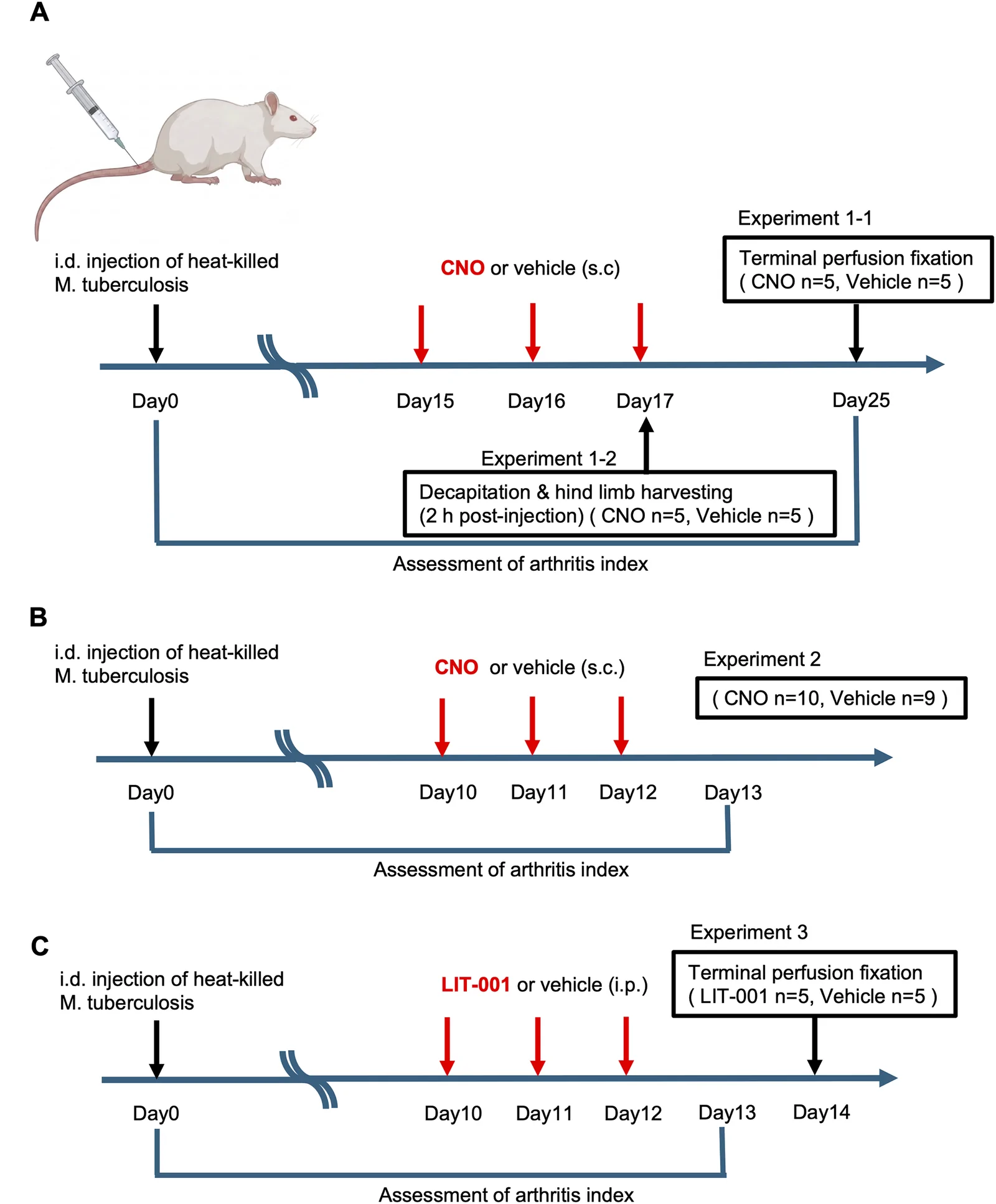
Schematic representation of the experimental schedule (Experiment 1, 2, and 3). Adjuvant arthritis (AA) was induced in rats by intradermal injection of heat-killed *Mycobacterium tuberculosis* (1 mg) into the base of the tail on Day 0. **(A)** Experiment 1 (Days 15–17 CNO intervention): CNO or vehicle (n = 10 each) was subcutaneously (s.c.) injected for three consecutive days from Day 15 to 17 post-induction. In Experiment 1-1 (n = 10; n = 5 each for CNO and vehicle), the arthritis score was evaluated until Day 25, followed by transcardial perfusion fixation. In Experiment 1-2 (another n = 10; n = 5 each for CNO and vehicle), rats were decapitated and hind paws harvested 2 h post-injection on Day 17. **(B)** Experiment 2 (Days 10–12 CNO intervention): CNO (n = 10) or vehicle (n = 9) was subcutaneously (s.c.) injected for three consecutive days from Day 10 to 12 post-induction. The arthritis score was evaluated until Day 13. **(C)** Experiment 3 (Days 10–12 LIT-001 intervention): LIT-001 or vehicle (n = 5 each) was intraperitoneally (i.p.) injected for three consecutive days from Day 10 to 12 post-induction. The arthritis score was evaluated until Day 13, and transcardial perfusion fixation was performed on Day 14. (M) tuberculosis, *Mycobacterium tuberculosis*; CNO, Clozapine N-oxide; s.c., subcutaneously; i.p., intraperitoneally.

#### 2.4.1 Effects of endogenous OXT neuron activation during the established phase of arthritis: long-term observation and histological evaluation

AA was induced in OXT-hM3Dq-mCherry Tg rats on Day 0 (**Figure 1A**). During the established phase of arthritis (Days 15–17), rats received daily subcutaneous injections of clozapine-N-oxide (CNO; 1 mg/kg; Sigma-Aldrich Japan Co. LLC, Tokyo, Japan) (19) or vehicle (physiological saline; Otsuka Pharmaceutical Factory, Tokushima, Japan) (n = 5 per group).

For evaluation of clinical signs and long-term disease progression, rats (n = 10) received CNO or vehicle, and body weight and arthritis index were measured daily throughout the experiment. On Day 25, animals were deeply anesthetized by an intraperitoneal injection of a mixture of three anesthetics containing midazolam (2.0 mg/kg), medetomidine (0.375 mg/kg), and butorphanol (2.5 mg/kg), followed by transcardial perfusion. Adequate anesthetic depth was confirmed by the absence of a withdrawal response to toe pinch before transcardial perfusion. Following tissue collection, death was confirmed by the absence of movement and heartbeat.

For histological evaluation, a separate group of rats (n = 10) received the same treatments. On Day 17, the rats were euthanized by decapitation without prior anesthesia 120 min after the final injection, and the hind limbs were collected for histological analysis. This method was chosen to avoid potential confounding effects of anesthetic agents on the experimental outcomes, including subsequent tissue analyses and potential future analyses of the collected blood samples. To minimize animal suffering, decapitation was performed by an experimenter experienced in the procedure. Death was confirmed by the absence of movement and heartbeat.

#### 2.4.2 Effects of endogenous OXT neuron activation during the onset phase of arthritis: evaluation of ankle swelling and mechanical hypersensitivity

AA was induced in OXT-hM3Dq-mCherry Tg rats (n = 19) on Day 0 (**Figure 1B**). Body weight and arthritis index were recorded daily throughout the experiment. Rats received daily subcutaneous injections of CNO (1 mg/kg, n = 10) or vehicle (n = 9) from Days 10 to 12.

To evaluate ankle swelling, the mediolateral and anteroposterior diameters of the ankle joint were measured using calipers on Day 10 before treatment and again on Day 13. Mechanical hypersensitivity was assessed using the Randall–Selitto test on Days 0, 10, and 13. On Day 13, following the completion of arthritis scoring and pain-related assessments, the rats were euthanized by carbon dioxide (CO₂) inhalation in a chamber measuring 30 × 35 × 40 cm (42 L). CO₂ was introduced at a flow rate of 15 L/min, corresponding to approximately 36% of the chamber volume per minute. Death was confirmed by the absence of respiration and heartbeat.

#### 2.4.3 Effects of the non-peptide OXT receptor agonist LIT-001 on adjuvant arthritis

AA was induced in Wistar rats (n = 10) on Day 0 (**Figure 1C**). Body weight and arthritis index were recorded daily throughout the experiment. Rats received intraperitoneal injections of the non-peptide OXT receptor agonist LIT-001 (10 mg/kg; MedChemExpress, Monmouth Junction, NJ, USA) (5, 20) or vehicle (1% sodium carboxymethyl cellulose [CMC-Na]; Nacalai Tesque, Kyoto, Japan) once daily from Days 10 to 12 (n = 5 per group).

Ankle swelling (Days 10 and 13) and mechanical hypersensitivity assessed by the Randall–Selitto test (Days 0, 10, and 13) were evaluated using the same protocol as in Experiment 2. On Day 14, animals were deeply anesthetized by intraperitoneal injection of a mixture of midazolam (2.0 mg/kg), medetomidine (0.375 mg/kg), and butorphanol (2.5 mg/kg), followed by transcardial perfusion. Adequate anesthetic depth was confirmed by the absence of a withdrawal response to toe pinch before transcardial perfusion. Following tissue collection, death was confirmed by the absence of movement and heartbeat.

### 2.5 Estimation of ankle joint cross-sectional area

The mediolateral and anteroposterior diameters of the ankle joint were measured using calipers. Assuming an elliptical shape, the ankle joint cross-sectional area was estimated using the following formula (21): S=(a/2)×(b/2)×π

where S represents the estimated area (mm²), a the mediolateral diameter (mm), and b the anteroposterior diameter (mm).

### 2.6 Measurement of mechanical nociceptive thresholds

Mechanical hypersensitivity was assessed using a Randall–Selitto analgesy meter (Analgesy-Meter, Ugo Basile, Gemonio, Italy) (22). Increasing mechanical pressure was applied to the dorsal surface of the right hind paw, and the force (g) at which the animal exhibited an escape response or vocalization was recorded as the mechanical nociceptive threshold. To avoid tissue damage, the cut-off value was set at 450 g. Measurements were repeated five times at each time point, and the mean of the three values remaining after exclusion of the highest and lowest values was used for analysis.

### 2.7 Histological evaluation of ankle joints

Ankle joints collected on Day 17 in Experiment 1 were fixed in 10% neutral buffered formalin for 48 h at room temperature and then decalcified in a rapid decalcifying solution (K-CX, Falma, Tokyo, Japan) at 4 °C for 48 h (23). Following decalcification, the tissues were neutralized with 5% sodium sulfate, embedded in paraffin, and sectioned sagittally at a thickness of 4 μm using a microtome.

Hematoxylin and eosin (H&E) staining was performed to assess inflammatory cell infiltration, synovial hyperplasia, and pannus formation. Toluidine blue staining was used to assess articular cartilage degeneration. Histological sections from all animals were examined under a light microscope (Nikon Corporation, Tokyo, Japan), and the histopathological findings were evaluated descriptively. Representative images are shown in **Figure 2**. No validated semi-quantitative histological scoring system was used, and the histopathological assessment was not performed in a blinded manner.

**Figure 2:**
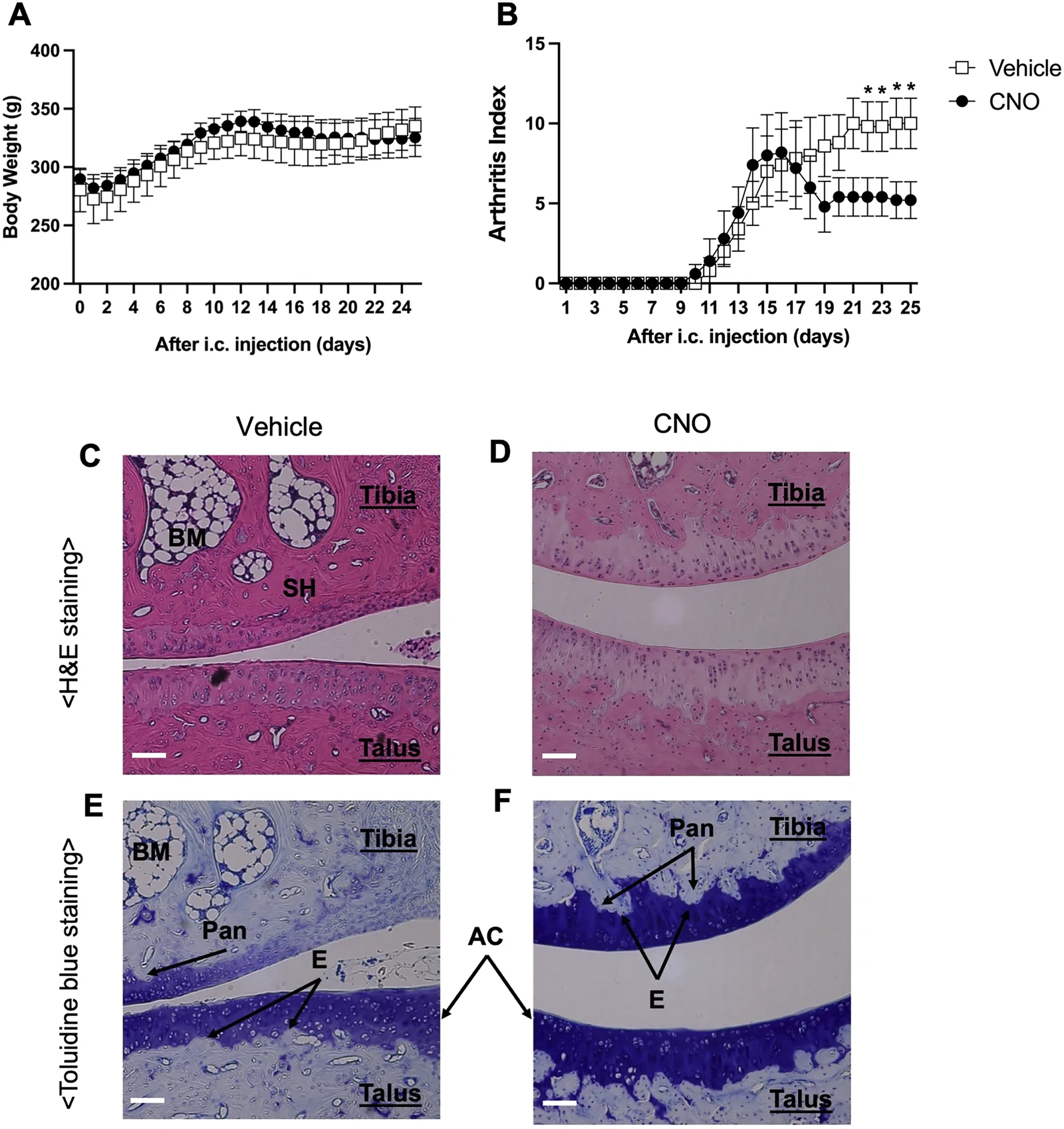
Effects of oxytocin neuron activation on arthritis severity and histopathological changes in AA rats. AA was induced by intradermal injection of heat-killed *M. tuberculosis* (1 mg) into the base of the tail. CNO (n = 5) or vehicle (n = 5) was injected subcutaneously (s.c.) from Day 15 to 17 after AA induction. **(A, B)** show data from Experiment 1-1, whereas panels C–F show representative ankle joint sections from a separate cohort in Experiment 1–2 collected on Day 17, 2 h after the final injection. **(A)** Body weight and **(B)** arthritis index were monitored throughout the experimental period. Data are presented as mean ± SEM. *P < 0.05 vs. vehicle group. Representative histopathological images of ankle joints obtained from vehicle- and CNO-treated AA rats are shown in **(C-F)**. Hematoxylin and eosin (H&E)-stained sections demonstrate marked synovial hyperplasia (SH), bone marrow (BM) involvement, and inflammatory changes in vehicle-treated rats **(C)**, whereas these pathological findings were less pronounced in CNO-treated rats **(D)**. Toluidine blue-stained sections show extensive pannus formation (Pan) and cartilage erosion **(E)** in vehicle-treated rats **(E)**, while joint architecture was relatively preserved in CNO-treated rats **(F)**. Scale bars = 100 μm. AA, adjuvant-induced arthritis; BM, bone marrow; CNO, clozapine-N-oxide; E, erosion site; H&E, hematoxylin and eosin; Pan, pannus; SH, synovial hyperplasia.

### 2.8 Brain tissue collection and section preparation

For immunohistochemical analyses, AA rats treated with LIT-001 or vehicle (n = 5 per group) were transcardially perfused with 0.1 M phosphate buffer (PB; pH 7.4) containing heparin (1,000 U/L), followed by 4% paraformaldehyde in 0.1 M PB. The brains were removed, post-fixed in the same fixative for 2 days, and sectioned coronally at 40 μm using a microtome (Yamato Kohki Industrial Co. Ltd, Saitama, Japan). Sections containing the paraventricular nucleus (PVN), supraoptic nucleus (SON), and dorsal raphe nucleus (DR) were used for subsequent analyses.

### 2.9 Fluorescence immunohistochemistry

Neuronal activation in the PVN, SON, and DR was evaluated by FosB immunoreactivity. Fluorescence immunohistochemistry was performed as described previously (16). Free-floating sections were incubated for 3 days at 4 °C with rabbit anti-FosB antibody (1:1000; Cat. No. ab184938, RRID: AB_2721123, Abcam, Cambridge, UK) diluted in phosphate-buffered saline (PBS; pH 7.6) containing 5% normal donkey serum and 0.3% Triton X-100.

To identify serotonergic neurons in the DR, rabbit anti-TPH antibody (1:200; Cat. No. bs-5601R, RRID: AB_11070339, Bioss Antibodies, Woburn, MA, USA) was used.

The secondary antibodies used were Alexa Fluor 546-conjugated goat anti-rabbit IgG (1:500; Cat. No. A-11035, RRID: AB_143051, Invitrogen, Thermo Fisher Scientific, Waltham, MA, USA) and Alexa Fluor 488-conjugated goat anti-rabbit IgG (1:1000; Cat. No. A-21206, RRID: AB_2535792, Invitrogen, Thermo Fisher Scientific, Waltham, MA, USA).

### 2.10 Image analysis and cell counting

Fluorescent images were captured using a fluorescence microscope (Nikon Corporation, Tokyo, Japan). For quantitative analysis, three consecutive sections containing the PVN, SON, or DR were selected from each animal. The numbers of FosB-immunoreactive (FosB-ir) cells and tryptophan hydroxylase-immunoreactive (TPH-ir) cells were counted, and the mean value from the three sections was calculated for each animal and used for statistical analysis.

### 2.11 Statistical analysis

Data are presented as mean ± SEM. Changes over time between groups, including body weight, arthritis index, ankle cross-sectional area, and mechanical nociceptive thresholds, were analyzed by two-way repeated-measures ANOVA followed by Šídák’s multiple-comparisons test. When the assumption of sphericity was violated, the Greenhouse–Geisser correction was applied. Differences in the numbers of FosB-ir cells and detectable TPH-ir cells between groups were analyzed using unpaired two-tailed Student’s t-tests.

All statistical analyses and graph preparation were performed using GraphPad Prism (Version 10.6.1 for Mac; GraphPad Software, Boston, MA, USA; RRID: SCR_002798). A P value < 0.05 was considered statistically significant.

## 3 Results

### 3.1 Activation of OXT neurons during the established phase of arthritis (days 15–17) reduced arthritis severity

Body weight increased until Day 12 in both the CNO-treated and vehicle-treated groups and then tended to decrease or plateau thereafter. No significant difference in body weight was observed between the groups throughout the observation period [F (25, 200) = 0.315, P = 0.999] (**Figure 2A**).

For the arthritis index, there was a significant interaction between time and treatment group [F (24, 192) = 2.419, P < 0.001] (**Figure 2B**). In both groups, arthritis scores increased from Day 10 and continued to rise until Day 16. Thereafter, scores continued to increase in the vehicle-treated group, whereas they declined from Day 17 onward in the CNO-treated group. Consequently, arthritis scores were significantly lower in the CNO-treated group than in the vehicle-treated group from Day 22 to Day 25 (Day 22, P = 0.034; Day 23, P = 0.034; Day 24, P = 0.024; Day 25, P = 0.024).

### 3.2 Effects of OXT neuron activation on histopathological changes in ankle joints of AA rats

Representative histological images are shown in **Figures 2C–F**. In the vehicle-treated group, synovial hyperplasia and inflammatory cell infiltration into the bone marrow cavity were observed, along with pannus formation within the joint space (**Figure 2C**). These changes were accompanied by apparent destruction of the articular cartilage and bone erosion (**Figure 2E**).

In contrast, the pathological changes appeared less pronounced in the CNO-treated group. Synovial hyperplasia and inflammatory cell infiltration appeared less prominent than in the vehicle-treated group, and less extensive pannus formation was observed (**Figure 2D**). Consistent with these findings, the articular cartilage and overall joint architecture appeared relatively well preserved in the CNO-treated group (**Figure 2F**).

### 3.3 Activation of OXT neurons during the onset phase of arthritis (days 10–12) suppressed ankle swelling and mechanical hypersensitivity

Body weight increased until Day 10 in both groups and then tended to decrease or plateau. No significant difference in body weight was observed between the groups throughout the experimental period [F(13, 221) = 0.215, P = 0.998] (**Figure 3A**).

**Figure 3:**
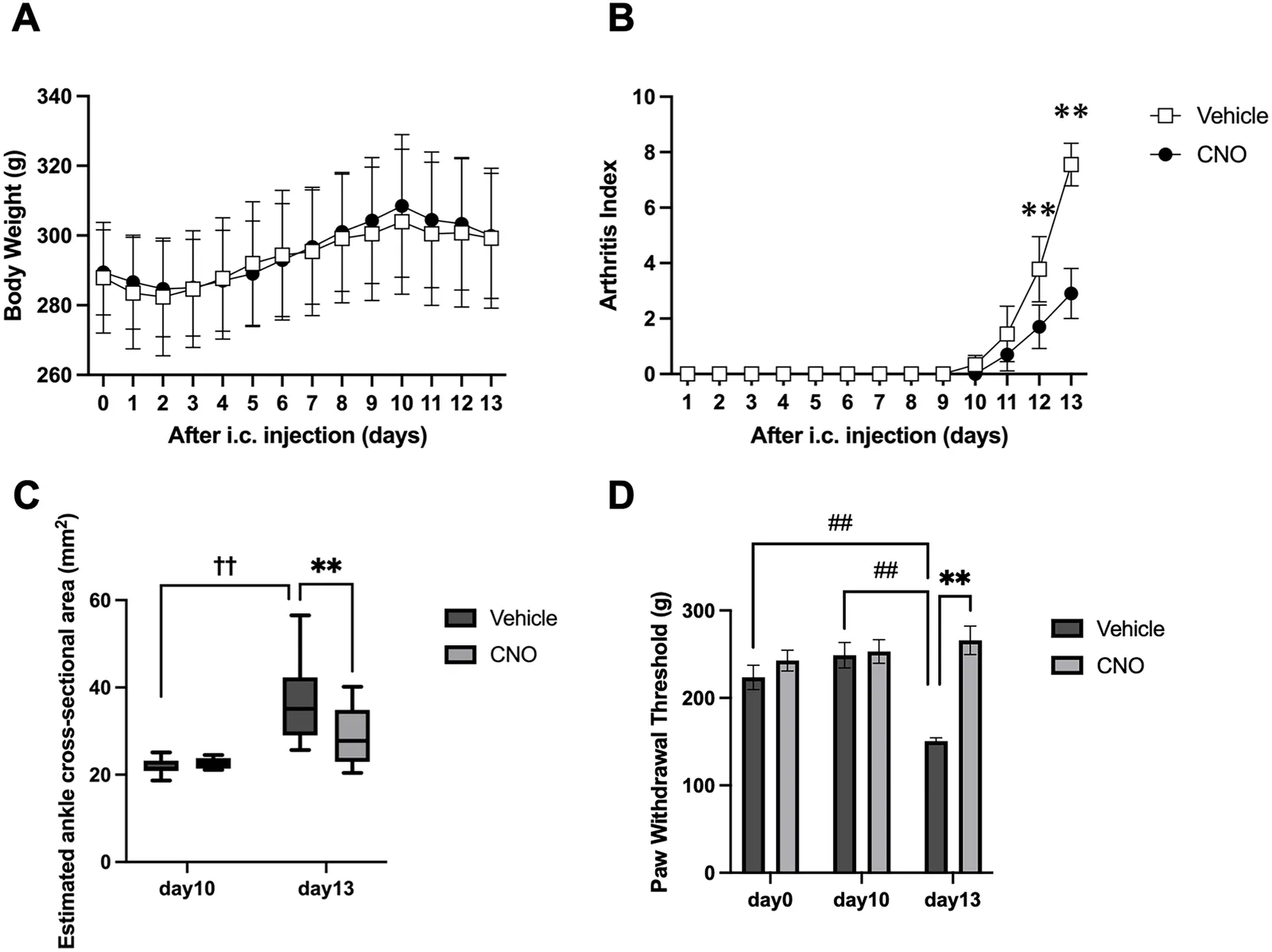
Effects of chemogenetic activation of oxytocin neurons on arthritis severity, ankle swelling, and mechanical hyperalgesia in AA rats. AA was induced by intradermal injection of heat-killed *Mycobacterium tuberculosis* (1 mg) into the base of the tail. CNO (n = 10) or vehicle (n = 9) was injected subcutaneously (s.c.) from Day 10 to Day 12 after AA induction. **(A)** Body weight and **(B)** arthritis index were monitored throughout the experimental period. **(C)** Estimated ankle cross-sectional area measured on Days 10 and 13 after AA induction. The estimated area was calculated as follows: (transverse diameter/2) × (anteroposterior diameter/2) × π. **(D)** Mechanical nociceptive thresholds were evaluated using the Randall–Selitto paw withdrawal test on Days 0, 10, and 13. Data are presented as mean ± SEM in **(A**, **B)**, and **(D)**, and as box-and-whisker plots in **(C)**. Statistical analyses were performed using two-way repeated-measures ANOVA followed by Šídák’s multiple comparisons test. **P < 0.01 vs. vehicle group; ††P < 0.01 vs. Day 10 within the same group **(C)**; ##P < 0.01 vs. Days 0 and 10 within the same group **(D)**. AA, adjuvant-induced arthritis; CNO, clozapine-N-oxide.

There was a significant interaction between time and treatment group for the arthritis index [F(12, 204) = 5.499, P < 0.001] (**Figure 3B**). Arthritis scores increased from Day 11 in the CNO-treated group and from Day 12 in the vehicle-treated group. In the vehicle-treated group, scores continued to rise thereafter, whereas the increase was suppressed after Day 12 in the CNO-treated group. As a result, the arthritis index was significantly lower in the CNO-treated group than in the vehicle-treated group on Day 13 (P < 0.001).

Ankle swelling was assessed by estimating the ankle joint cross-sectional area before treatment (Day 10) and after three consecutive days of treatment (Day 13) (**Figure 3C**). No significant difference was observed between the groups on Day 10. In the vehicle-treated group, the ankle joint cross-sectional area was significantly increased on Day 13 compared with Day 10. In contrast, the ankle joint cross-sectional area was significantly smaller in the CNO-treated group than in the vehicle-treated group on Day 13 (P = 0.008), indicating reduced ankle swelling.

Mechanical nociceptive thresholds were assessed using the Randall–Selitto test (**Figure 3D**). Measurements were obtained before AA induction (Day 0), before treatment (Day 10), and after three consecutive days of treatment (Day 13). In the vehicle-treated group, the nociceptive threshold was significantly lower on Day 13 than on Day 10 (P < 0.001). In contrast, no significant change was observed between Days 10 and 13 in the CNO-treated group.

### 3.4 Effects of LIT-001 on arthritis severity, ankle swelling, and mechanical hypersensitivity in AA rats

Body weight increased over time in both groups. Body weight increased until approximately Day 10 in the vehicle-treated group and until approximately Day 9 in the LIT-001-treated group, after which it remained relatively stable (**Figure 4A**). No significant Treatment × Time interaction was observed for body weight [F(1.242, 7.451) = 0.408, P = 0.586].

**Figure 4:**
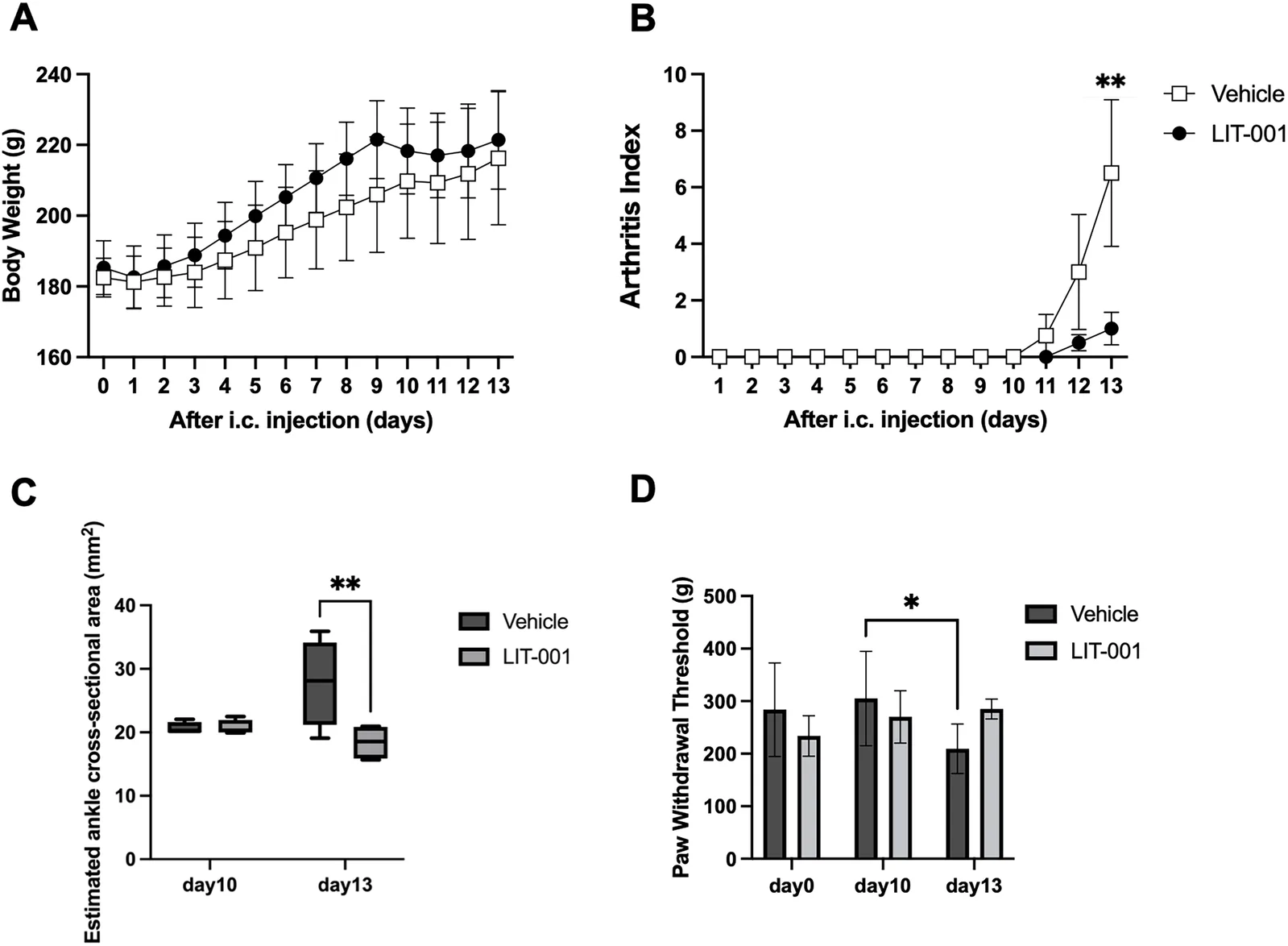
Effects of LIT-001 on arthritis severity, ankle swelling, and mechanical hyperalgesia in AA rats. AA was induced by intradermal injection of heat-killed *Mycobacterium tuberculosis* (1 mg) into the base of the tail. LIT-001 or vehicle was administered intraperitoneally (i.p.) from Days 10 to 12 after AA induction. **(A)** Body weight and **(B)** arthritis index were monitored throughout the experimental period. **(C)** Estimated ankle cross-sectional area measured on Days 10 and 13 after AA induction. The estimated area was calculated as follows: (transverse diameter/2) × (anteroposterior diameter/2) × π. **(D)** Mechanical nociceptive thresholds were evaluated using the Randall–Selitto paw withdrawal test on Days 0, 10, and 13. Data are presented as mean ± SEM in **(A**, **B**, **D)**, and as box-and-whisker plots in **(C)**. Statistical analyses were performed using two-way repeated-measures ANOVA followed by Šídák’s multiple comparisons test. *P < 0.05 vs. Day 10 within the same group; **P < 0.01 vs. vehicle group. AA, adjuvant-induced arthritis.

There was a significant interaction between time and treatment group for the arthritis index [F(12, 72) = 3.043, P = 0.002] (**Figure 4B**). In the vehicle-treated group, arthritis scores increased from Day 11 and continued to rise thereafter. In contrast, this increase was markedly attenuated in the LIT-001-treated group. Arthritis scores were significantly lower in the LIT-001-treated group than in the vehicle-treated group on Day 13 (P < 0.001).

Ankle swelling was assessed by estimating the ankle joint cross-sectional area before treatment (Day 10) and on Day 13 (**Figure 4C**). No significant difference was observed between the groups on Day 10. In the vehicle-treated group, the ankle joint cross-sectional area increased significantly by Day 13. In contrast, the ankle joint cross-sectional area was significantly smaller in the LIT-001-treated group than in the vehicle-treated group on Day 13 (P = 0.004).

Mechanical nociceptive thresholds were assessed using the Randall–Selitto test (**Figure 4D**). In the vehicle-treated group, the nociceptive threshold was significantly lower on Day 13 than on Day 10 (P = 0.039). In contrast, no significant change was observed between Days 10 and 13 in the LIT-001-treated group.

### 3.5 Effects of LIT-001 on FosB expression in the PVN, SON, and DR

To examine the effects of LIT-001 on central neuronal activity, brain tissues were collected on Day 14 in Experiment 3, and the numbers of FosB-immunoreactive (FosB-ir) cells in the PVN, SON, and DR were quantified. Representative images are shown in **Figure 5A**.

**Figure 5:**
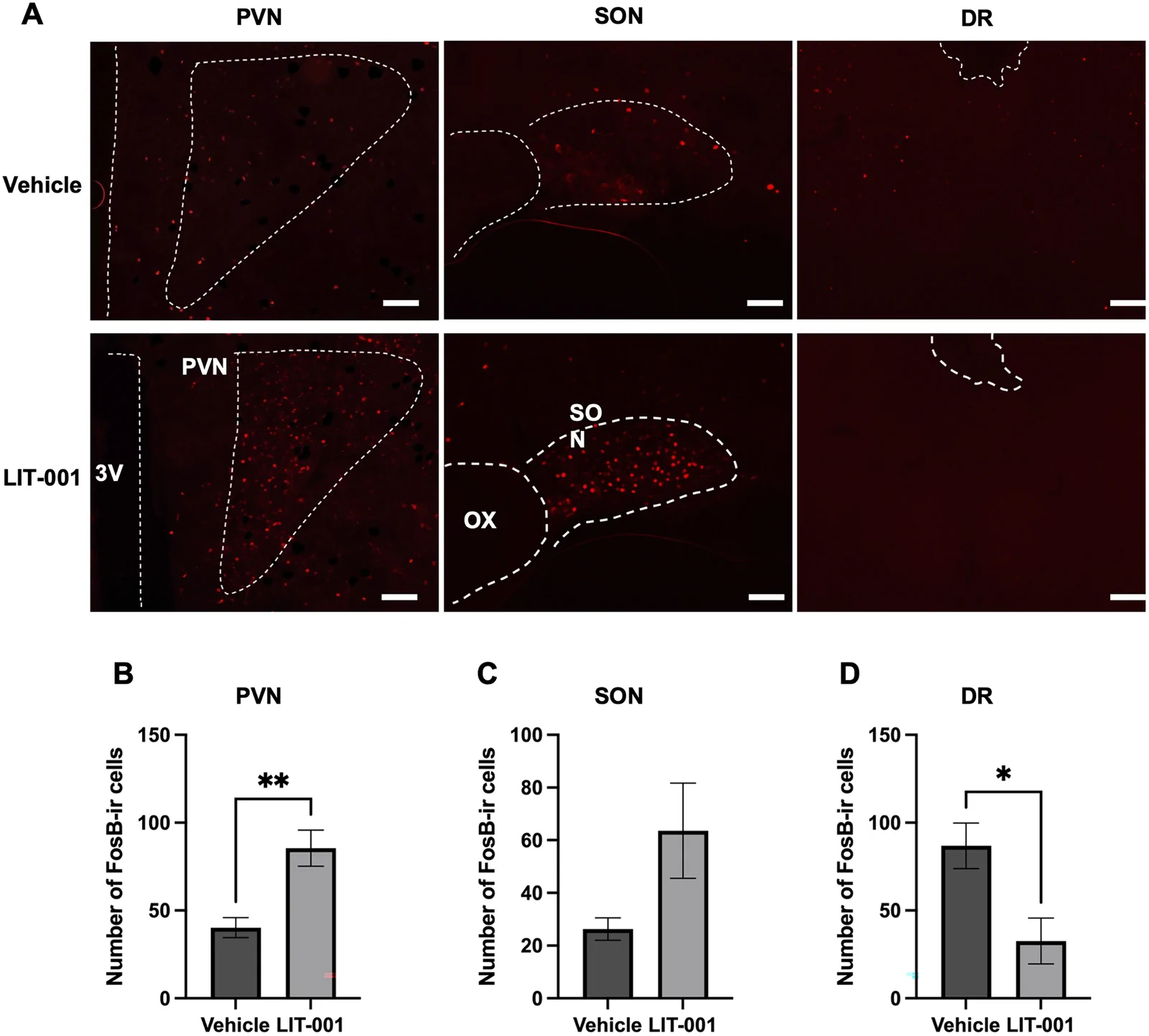
Effects of LIT-001 on FosB expression in the PVN, SON, and DR. **(A)** Representative microphotographs showing FosB-immunoreactive cells in the paraventricular nucleus of the hypothalamus (PVN), supraoptic nucleus (SON), and dorsal raphe nucleus (DR) of AA rats treated with vehicle or LIT-001. Quantitative analysis of the number of FosB-ir cells per section in the PVN **(B)**, SON **(C)**, and DR **(D)** (n = 5 each). Data are presented as mean ± SEM. Statistical comparisons between the vehicle and LIT-001 groups were performed using an unpaired two-tailed Student’s t-test. *P < 0.05, **P < 0.01 vs. vehicle group. Scale bars = 100 μm. AA, adjuvant-induced arthritis; PVN, paraventricular nucleus of the hypothalamus; SON, supraoptic nucleus; DR, dorsal raphe nucleus; 3V, third ventricle; OX, Optic Chiasm.

The number of FosB-ir cells in the PVN was significantly greater in the LIT-001-treated group than in the vehicle-treated group (P = 0.008) (**Figure 5B**). In the SON, no significant difference in the number of FosB-ir cells was observed between the groups (P = 0.108) (**Figure 5C**). In the DR, the number of FosB-ir cells was significantly lower in the LIT-001-treated group than in the vehicle-treated group (P = 0.019) (**Figure 5D**).

### 3.6 Effects of LIT-001 on TPH-immunoreactivity in the DR

To examine the effects of LIT-001 on TPH immunoreactivity in the DR, immunofluorescence staining for tryptophan hydroxylase (TPH) was performed. Representative images are shown in **Figure 6A**. TPH-immunoreactive (TPH-ir) cells were observed in both groups.

Quantitative analysis showed that the number of detectable TPH-ir cells in the DR was significantly lower in the LIT-001-treated group than in the vehicle-treated group (P = 0.005) (**Figure 6B**).

**Figure 6:**
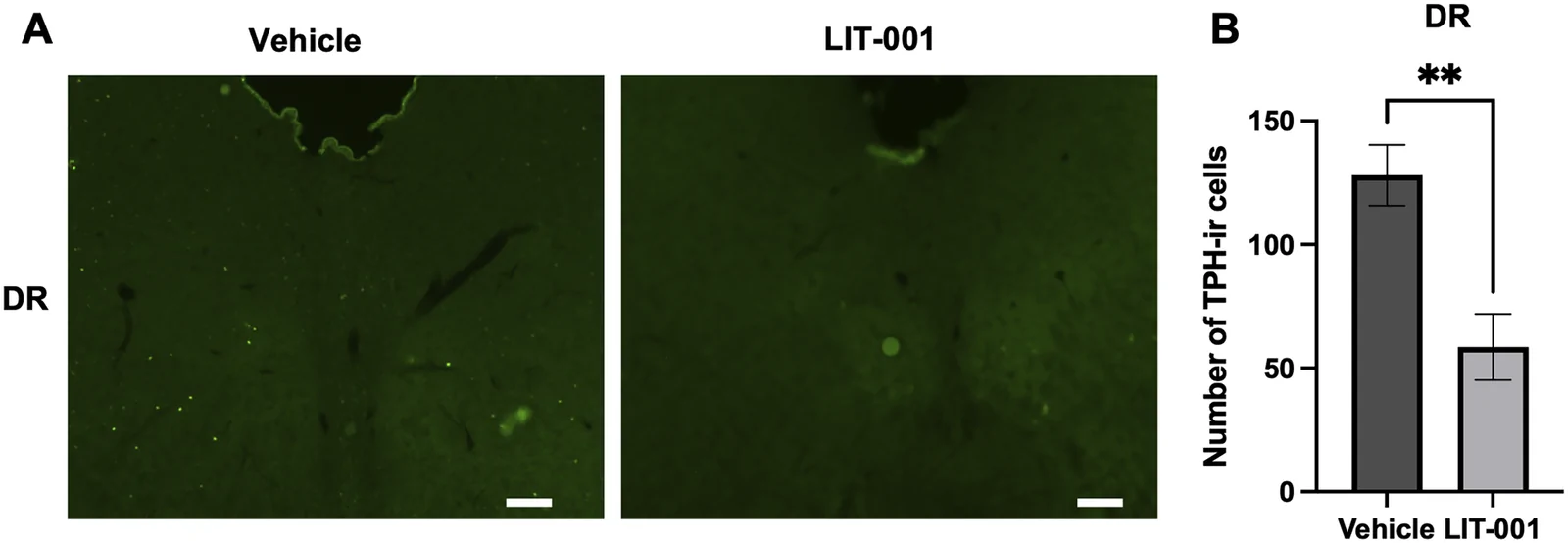
Effect of LIT-001 on TPH-ir cells in the dorsal raphe nucleus (DR). **(A)** Representative immunofluorescence images of tryptophan hydroxylase-immunoreactive (TPH-ir) cells (green) in the DR of the vehicle-treated group (left) and LIT-001-treated group (right). Scale bar = 100 μm. **(B)** Quantitative comparison of the number of TPH-ir cells between the vehicle and LIT-001 groups.Data are expressed as mean ± SEM (n = 5 per group). Statistical comparisons between the vehicle and LIT-001 groups were performed using an unpaired two-tailed Student’s t-test. **P < 0.01 vs. vehicle group (Student’s *t*-test). TPH-ir, tryptophan hydroxylase-immunoreactive; DR, dorsal raphe nucleus.

## 4 Discussion

We found that activation of the endogenous OXT system attenuated arthritis pathology in AA rats. Both chemogenetic activation of OXT neurons using DREADDs and pharmacological stimulation of OXT receptors with LIT-001 improved arthritic symptoms and pain-related behaviors. In OXT-hM3Dq-mCherry Tg rats, CNO administration during the established phase of arthritis (Days 15–17) suppressed the progression of arthritis scores, while histological examination showed less pronounced inflammatory cell infiltration, synovial hyperplasia, pannus formation, cartilage destruction, and bone erosion in the ankle joints. Likewise, activation of OXT neurons during the onset phase of arthritis (Days 10–12) attenuated the increase in arthritis scores, ankle swelling, and the decline in mechanical nociceptive thresholds. Repeated administration of the non-peptide OXT receptor agonist LIT-001 also reduced arthritis severity, ankle swelling, and mechanical hypersensitivity.

In the AA model used here, vehicle-treated animals developed progressive arthritis after Days 10–12, accompanied by ankle swelling, reduced mechanical nociceptive thresholds, and histological joint damage. These features are consistent with the well-characterized course of chronic inflammatory arthritis in AA rats (13, 14). The effects of LIT-001 were directionally similar to those observed after chemogenetic activation of OXT neurons, although the two interventions were tested in different experimental settings and should not be directly compared. These findings support the involvement of OXT signaling in the regulation of arthritis pathology.

Interestingly, the effects of OXT neuron activation differed according to the timing of intervention. When CNO was administered during the onset phase of arthritis (Days 10–12), increases in arthritis scores, ankle swelling, and mechanical hypersensitivity were suppressed, suggesting that activation of the OXT system limited the progression of arthritis during disease development. In contrast, administration during the established phase of arthritis (Days 15–17) attenuated the further progression of arthritic symptoms that had already developed. However, because the observation periods differed between the onset- and established-phase experiments, the magnitude and durability of the effects between these intervention periods cannot be directly compared. In addition, the onset-phase experiment did not include long-term histological assessment, and therefore it remains unclear whether the early intervention produced sustained protection against joint pathology. These findings suggest that activation of the OXT system may influence arthritis during both the early and established phases of disease progression, although further studies with comparable observation periods are needed to determine whether the effects differ according to the timing of intervention.

The beneficial effects of OXT signaling may, at least in part, be mediated through modulation of peripheral inflammation. OXT has been reported to regulate inflammatory responses, and OXT receptor expression changes in immune cells and inflamed tissues under inflammatory conditions (12). In addition, OXT receptor expression has been identified in joint tissues, and links between the OXT system and joint diseases have been described previously (10). In the present study, activation of OXT neurons reduced ankle swelling, and the histological findings were consistent with less pronounced joint pathology. Together, these observations indicate that activation of the OXT system is involved in limiting local inflammatory changes within the joint.

These findings may also be considered within the broader framework of neuroimmune regulation, including the emerging concept of the inflammatory reflex. The inflammatory reflex refers to the neural regulation of peripheral immune responses and has been implicated in the control of excessive inflammation through autonomic pathways (24, 25). In experimental arthritis, vagal stimulation has been shown to suppress joint inflammation through neural pathways involving the brainstem and hypothalamic circuits, including the PVN (26). In addition, accumulating evidence suggests functional interactions between the OXT system and vagal pathways. Peripheral OXT can activate vagal afferent neurons and engage the nucleus tractus solitarius (NTS), while vagal afferent signaling can activate OXT neurons in the PVN (27). Although the present study did not directly examine vagal activity or the involvement of vagal pathways, these findings raise the possibility that interactions between the OXT system and autonomic neural circuits may contribute to the regulation of inflammation and pain in arthritis. Further studies are needed to determine whether vagal pathways contribute to the effects of OXT signaling observed in the present study.

The OXT system could also influence pain processing at the spinal and supraspinal level. OXT neurons originating in the PVN project to the spinal dorsal horn (2, 28), where OXT modulates nociceptive transmission through effects on excitatory synaptic inputs and inhibitory interneurons (29). In addition, recent evidence has identified a parvocellular OXT pathway from the PVN to the ventrolateral periaqueductal gray (vlPAG), an important component of the descending pain modulatory system. Activation of this pathway suppresses nociceptive responses in spinal neurons and produces analgesic effects in both inflammatory and neuropathic pain models (28). In the present study, both CNO and LIT-001 prevented the decline in mechanical nociceptive thresholds observed in vehicle-treated animals. These findings suggest that the beneficial effects of OXT signaling may involve not only suppression of peripheral inflammation but also modulation of central pain-processing pathways. However, neuronal activity in the spinal dorsal horn and nucleus tractus solitarius (NTS) was not directly assessed in the present study. Therefore, although our findings are consistent with a possible involvement of spinal nociceptive processing and descending pain modulatory pathways, we cannot determine whether these pathways are directly engaged by OXT signaling. Future studies assessing neuronal activity markers such as pERK and c-Fos in the spinal dorsal horn and NTS will be important for further elucidating the neural circuits underlying the analgesic effects of OXT in inflammatory arthritis.

The use of exogenous OXT has several limitations, including restricted blood–brain barrier permeability, a short biological half-life, and limited receptor selectivity (30). Moreover, at high concentrations, OXT can activate vasopressin V1a receptors and TRPV1 channels in addition to OXT receptors (31, 32). As a result, studies based solely on exogenous OXT administration do not readily distinguish the physiological functions of endogenous OXT signaling. By using DREADDs, we chemogenetically activated endogenous OXT neurons and examined their potential contribution to chronic inflammatory pain. The present findings support the view that endogenous OXT signaling participates in the regulation of chronic inflammatory pain.

To complement the chemogenetic approach, we also evaluated the non-peptide OXT receptor agonist LIT-001 (20). Previous studies have shown that LIT-001 produces antinociceptive effects in inflammatory pain models (5). Consistent with those reports, repeated administration of LIT-001 reduced arthritis severity, ankle swelling, and mechanical hypersensitivity in AA rats. These data provide pharmacological support for the results obtained using DREADDs and further support a role for OXT receptor signaling in the attenuation of arthritis pathology.

Analysis of FosB expression following LIT-001 treatment revealed distinct responses in the PVN and DR. FosB-immunoreactive cell numbers increased in the PVN, whereas no significant change was detected in the SON. Although both nuclei contain OXT-producing neurons, their projection patterns and physiological roles differ. OXT neurons in the SON primarily regulate hormone release into the systemic circulation through projections to the posterior pituitary (3). In contrast, the PVN sends projections not only to the posterior pituitary but also to the spinal dorsal horn and brainstem, where it participates in pain modulation and autonomic regulation (2). In this context, the increase in FosB expression observed in the PVN may reflect altered neuronal activity associated with the improvement of inflammatory responses and pain-related behaviors following OXT receptor stimulation.

In the DR, FosB-immunoreactive cell counts and the number of detectable TPH-immunoreactive cells were lower in LIT-001-treated animals than in vehicle-treated controls. Because LIT-001 also improved arthritis severity and pain-related behaviors, these reductions may reflect decreased neuronal activity secondary to reduced peripheral inflammation and diminished nociceptive input. Alternatively, previous studies have demonstrated projections from the PVN to the DR and the presence of OXT receptors within the DR (33, 34). It is therefore possible that OXT signaling directly influences brainstem monoaminergic circuits through actions on the DR. The present findings raise the possibility that the OXT system contributes to the regulation of pain-related neural circuits involving the DR.

Several limitations should be considered when interpreting these findings. First, we did not directly assess circulating or local OXT levels, OXT receptor expression in joint tissues, or the brain penetration and sites of action of LIT-001. In addition, OXT receptor antagonists and local pharmacological interventions were not used. Consequently, the observed effects cannot be definitively attributed to OXT receptor-dependent mechanisms, nor can the primary sites of action be identified. The anatomical connections between OXT neurons and peripheral joint tissues were not examined. Future studies using anatomical tracing approaches will be important to determine whether OXT neurons have direct or indirect connections with peripheral joint tissues and to clarify their potential contribution to the regulation of arthritis. Second, although the numbers of FosB-immunoreactive cells in the PVN and DR and detectable TPH-immunoreactive cells in the DR differed between groups, the causal relationship between these immunohistochemical findings and pain regulation was not directly examined. Likewise, the functional contributions of PVN OXT neurons and DR circuits remain unclear. It is also uncertain whether the changes observed in the DR resulted from direct effects of OXT signaling or occurred secondary to reduced inflammation and nociceptive input. Further studies assessing neuronal activity and circuit function will be needed to clarify these relationships. Third, animals were not randomly assigned to the treatment groups, and the evaluators were not blinded to treatment allocation when assessing arthritis scores, mechanical nociceptive thresholds, or histopathological findings. Therefore, the possibility of observer bias cannot be completely excluded. In addition, a CNO-treated non-DREADD control group was not included, and thus potential DREADD-independent effects of CNO, including those related to its conversion to clozapine, cannot be completely excluded (35, 36). In separate experiments conducted in our laboratory using non-Tg Wistar rats, we observed no significant difference in Fos expression between saline- and CNO-treated animals, or between saline-treated non-Tg Wistar rats and Tg rats (37). These findings provide some supporting evidence that CNO administration did not markedly alter neuronal Fos expression under these experimental conditions. However, because these experiments were conducted using an AVP-DREADD model, they cannot substitute for a CNO-treated non-DREADD control in the present OXT-DREADD experiments. Future studies including an appropriate CNO-treated non-DREADD control group will therefore be important to further establish the specificity of the effects observed following chemogenetic activation of OXT neurons. Furthermore, although the effects of CNO and LIT-001 were evaluated using multiple outcome measures, including arthritis scores, ankle swelling, and mechanical nociceptive thresholds, behavioral specificity tests such as the open field or elevated plus maze were not performed. Thus, potential confounding effects of motor impairment, sedation, or altered anxiety-related behavior on the behavioral outcomes cannot be completely excluded. Future studies incorporating these behavioral assessments will be important to further clarify the interpretation of the observed effects. Finally, histological evaluation was descriptive and was not performed using a validated scoring system or blinded assessment. Therefore, these findings should be interpreted cautiously. Future studies using blinded assessment and quantitative histological scoring will be needed to confirm the effects of OXT system activation on joint pathology.

In summary, both activation of endogenous OXT neurons and pharmacological stimulation of OXT receptors ameliorated arthritis severity and pain-related behaviors in AA rats, while histological examination revealed less pronounced joint pathological changes following OXT system activation. These findings support a role for the OXT system in the regulation of chronic inflammatory pain and suggest that OXT signaling may contribute to the modulation of arthritis and pain through neuroimmune interactions. Further studies are needed to determine whether these findings have clinical relevance to inflammatory arthritis.

## Data Availability

The raw data supporting the conclusions of this article will be made available by the authors, without undue reservation.
